# 

**Published:** 1891-01

**Authors:** 


### TABLE OF CONTENTS.

ORIGINAL COMMUNICATIONS. PAGE

The Influence of Adenoid Hypertrophy at the Vault of the Pharynx upon the Development of the Hard Palate. By Dr. D. Bryson Delavan, New York . . . . . . . . . . . . . . . . . . . . . . . . . 1

Concerning Third Molars. By Dr. C. A. Brackett, Newport, R. I. . . 10

Professional Ethics. By Dr. S. B. Bartholomew, Springfield, Mass. . 17

The Relation of Dental Societies to the Profession. By Jos. King Knight, D.D.S., Boston, Mass. . . . . . . . . . . . . . . . . . . 23

A Case of Necrosis of the Superior Maxillary Bone. By B. D. Friedenwald, D.D.S. . . . . . . . . . . . . . . . . . . . . . . . . . 29

REPORTS OF SOCIETY MEETINGS.

American Dental Association.—Thirtieth Annual Meeting, Excelsior Springs, Mo., August 5 to 8, 1890 . . . . . . . . . . . . . . . . . 31

New York Odontological Society . . . . . . . . . . . . . . . . . . . 41

American Academy of Dental Science . . . . . . . . . . . . . . . . . 55

Union Dental Meeting, held in Boston, Massachusetts, October 28 to 31, 1890 . . . . . . . . . . . . . . . . . . . . . . . . . . . . 63

EDITORIAL.

American Dental Degrees in England . . . . . . . . . . . . . . . . 66

Koch's Treatment of Tuberculosis . . . . . . . . . . . . . . . . . . 68

Bibliography . . . . . . . . . . . . . . . . . . . . . . . . . . . . 69

OBITUARY.

William W. Fouché, D.D.S. . . . . . . . . . . . . . . . . . . . . . 77

CURRENT NEWS . . . . . . . . . . . . . . . . . . . . . . . . . . . 78

